# EP26 HIV infection masquerading as connective tissue disease

**DOI:** 10.1093/rap/rkaa052.025

**Published:** 2020-11-03

**Authors:** Zeeshan Javaid, Babitha Mekkayil, Anupam Paul

**Affiliations:** r1 Darlington Memorial Hospital, Darlington, United Kingdom; r2 The James Cook University Hospital, Middlesbrough, United Kingdom

## Abstract

**Case report - Introduction:**

Connective tissue diseases are multisystem disorders. Diagnosis and evaluation of suspected cases is not straight forward in most of the cases. This case describes the significance of considering a broader approach when evaluating a suspected case of connective tissue disease.

**Case report - Case description:**

We describe a case of 58 years old non-smoker lady, presented with acute onset livedo reticularis rash on lower limbs and background of sicca symptoms, oral ulcers, fatigue, paraesthesias in feet and arthralgias without any systemic or inflammatory joint symptoms.

General examination showed livedo reticularis rash on both elbows and lower legs. There was no evidence of peripheral joint synovitis, but she had nodal osteoarthritis in her hands. Systemic examination was unremarkable.

Investigations revealed anaemia, pancytopenia, ESR of 77, low C4 and urine dipstick positive for leucocytes, nitrates, protein, and blood. Schirmer’s test, ANA and ENA screen was positive with positive RNP and SMdp antibody. She also had hypergammaglobulinemia in a polyclonal pattern. Nerve Conduction and EMG studies revealed mild axonal sensory neuropathy.

**Case report - Discussion:**

This lady appeared to have mixed connective tissue disease with mixed features of Sjögren’s syndrome and systemic lupus erythematosus. She was started on Hydroxychloroquine but stopped it shortly after developing floaters in her eye. She had poor response to Depomedrone injection. She had ongoing symptoms of fatigue, weight-loss, loose stools, and abdominal pain, investigated further and CT scan showed hyperdense liver lesions and mesenteric lymphadenopathy. Esophagogastroduodenoscopy showed oesophageal candidiasis. She was admitted with progressive symptoms. Further investigations showed a positive HIV test and liver biopsy came back positive for anaplastic lymphoma, later she was diagnosed with advanced HIV disease, rapidly deteriorated with neutropenic sepsis and multi-organ failure, and unfortunately died.

**Case report - Key learning points:**

This lady initially presented with symptoms of connective tissue disease and investigations in keeping with this diagnosis. Unfortunately, by the time she was tested for HIV infection, it was already too late. There could be overlap of symptoms of connective tissue disease and viral infections e.g. HIV infection. Autoantibodies may be falsely positive in infections e.g. HIV and in malignancy. Risk factors of HIV infection should be considered during assessment of multisystem diseases like connective tissue diseases particularly prior to immunosuppression. Viral screening including HIV test should be considered in all high-risk patients and particularly if the symptoms are atypical and do not quite fit well with the diagnosis.

